# A Review on Electrochemical Sensors and Biosensors Used in Phenylalanine Electroanalysis

**DOI:** 10.3390/s20092496

**Published:** 2020-04-28

**Authors:** Ancuta Dinu, Constantin Apetrei

**Affiliations:** Department of Chemistry, Physics and Environment, Faculty of Sciences and Environment, “Dunărea de Jos” University of Galaţi, 47 Domneasca Street, 800008 Galaţi, Romania; ancuta.dinu@ugal.ro

**Keywords:** phenylalanine, sensor, biosensor, electrochemistry, phenylketonuria

## Abstract

Phenylalanine is an amino acid found in breast milk and in many foods, being an essential nutrient. This amino acid is very important for the human body because it is transformed into tyrosine and, subsequently, into catecholamine neurotransmitters. However, there are individuals who were born with a genetic disorder called phenylketonuria. The accumulation of phenylalanine and of some metabolites in the body is dangerous and may cause convulsions, brain damage and mental retardation. Determining the concentration of phenylalanine in different biologic fluids is very important because it can provide information about the health status of the individuals envisaged. Since such determinations may be made by using electrochemical sensors and biosensors, numerous researchers have developed such sensors for phenylalanine detection and different sensitive materials were used in order to improve the selectivity, sensitivity and detection limit. The present review aims at presenting the design and performance of some electrochemical bio (sensors) traditionally used for phenylalanine detection as reported in a series of relevant scientific papers published in the last decade.

## 1. Introduction

Mental retardation, vitiligo and depression, in particular, are conditions which have increased in the last decade, proving to originate, in numerous cases, in hereditary metabolic diseases, such as phenylketonuria (PKU), also called hyperphenylalaninemia [1]. The researchers’ special interest in the treatment of this disease is illustrated by the present study which approaches the most relevant studies regarding the determination of phenylalanine (Phe) level in pharmaceuticals and in biologic samples by using high performance liquid chromatography or gas chromatography, mass spectrometry, fluorescent methods and electrochemical methods made in the last 10 years.

According to numerous studies in the field, the level of Phe may be detected in human beings as early as the neonatal period, being part of the first sets of analyses performed on newborns. The oldest method for determining the level of Phe [2,3] in patients diagnosed with diseases conditioned by this amino acid implied taking and analyzing blood or urine samples and making bacterial inhibition tests for Phe detection.

PKU is due to an enzyme genetic defect which favors the accumulation of a significant amount of Phe and of its metabolizing products (i.e., phenylpyruvic acid, phenylacetic acid and phenyllactic acid) in the bloodstream. The high concentration of Phe inhibits another enzyme which decreases melanin synthesis (a pigment in the skin) [4,5].

The means of preventing PKU, the treatments which may be used in order to obtain optimal results and the means of detecting this metabolic disease accurately and in due time are still highly debated issues. Out of the numerous methods commonly used for determining Phe, those based on the use of sensors and biosensors and on electrochemical analyses, respectively, remain of great interest, because they are the fastest, the most reliable and the most inexpensive methods. In addition, they may represent the basis for the development of new-generation technology [6,7].

## 2. Phenylalanine. Properties and Importance for the Human Body

**Figure 1:**
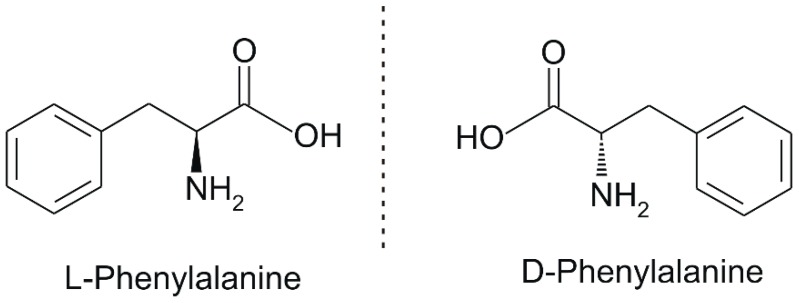
Chemical structures of Phe stereoisomers.

Phe, which was discovered in the yellow lupine by Schulze and Barbieri in 1879 and synthesized by Erlenmeyer and Lipp three years later, is an aromatic *α*-amino acid of the chemical formula C_9_H_11_NO_2_ (Figure 1). It is relatively nonpolar (hydrophobic) and it can participate in hydrophobic interactions [8].

Phe comes in three possible forms, namely L-Phe (natural), D-Phe (synthesized) and DL-Phe (found in nutritional supplements). It is converted to tyrosine in the body and it is used in the biosynthesis of two important neurotransmitters, dopamine and norepinephrine [9].

From a biologic point of view, Phe is an essential amino acid for human development being used as a precursor in the synthesis of tyrosine and of other compounds which contain a six-membered aromatic ring, such as: dopamine and compounds known as catecholamines: epinephrine (adrenaline), norepinephrine (norepinephrine) and melanin (skin pigment) [10,11]. It is also used to reduce hunger pains and depression and to improve memory [12].

A study regarding the Parkinson’s disease conducted on animals suggests that Phe is capable of improving walking disabilities, stiffness, speech difficulties and depression caused by this disease. D-Phe was shown to stimulate natural opioids—beta endorphins and encephalins—which play a central role in pain management [9,13].

Last, but not least, since a diet lacking Phe, tyrosine, tryptophan may increase irritability, thus affecting mood, it is assumed that Phe is effective in treating depression. 80%–90% of the cases included in a clinical study made on patients identified with low Phe levels in their urine and blood responded positively to the treatment of depression by using this amino acid. The dose of Phe for treating depression in adults generally ranged between 1–10 g/day which corresponds to a range of 11.6–116 mg/kg/day [14].

Studies were also conducted on the use pf Phe in treating children diagnosed with the attention deficit hyperactivity disorder (ADHD) triggered by low dopamine levels. The use of DL-Phe improved some of the symptoms of this condition: more precisely anger, restlessness and poor concentration, but only for a three-month period, the beneficial effects disappearing after this interval [15].

L-Phe is a potent satiety hormone releasing agent (cholecystokinin—CCK) and various studies, mainly performed on men, show that L-Phe reduces energy intake.

In nature, L-Phe is found in mammalian breast milk, but this amino acid may also be assimilated from plant and soy products (seeds and nuts offer about 2.5 g of Phe to 200 calories), from animal products (e.g., eggs, seafood, meat and cheese which provide 2–3 g of Phe to 200 calories) or from drug supplements available either as tablets of various concentrations—375 mg, 500 mg, 750 mg and 1000 mg or as powder [10].

**Figure 2:**
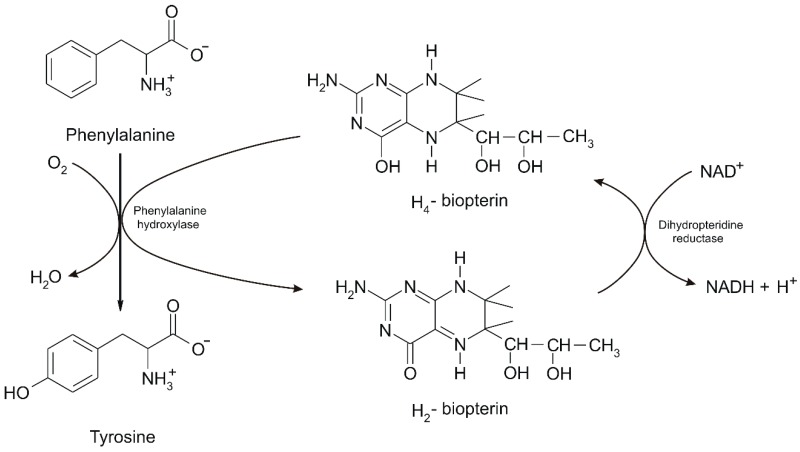
Transformation of L-Phe into L-Tyr biocatalyzed by PAH [1].

Being an essential amino acid, Phe cannot be produced by the body, which means that it must be ingested in the daily diet. Phenylalanine hydroxylase (PAH) is synthesized in the liver and kidneys and mutations in the gene expressing PAH may lead to PKU, the serious metabolic disease mentioned above. PAH, the enzyme which metabolizes excess Phe, has a mixed oxidase function because it incorporates an oxygen atom into the hydroxyl product, respectively tyrosine and another oxygen atom into the water. Its coenzyme is tetrahydrobiopterin (BH_4_) which donates two hydrogen atoms in the PAH reaction, while BH_4_ is in a lesser state called dihydrobiopterin (BH_2_) after donating two hydrogen atoms. BH_2_ is converted back to BH_4_ by dihydrobiopterin reductase with NADH + H^+^ as coenzyme [1] (Figure 2).

Over 400 gene mutations for PAH are known in the case of children, which lead to an active or inactive part of PAH and generate PKU. In this disease, Phe cannot be transformed into tyrosine, thus increasing the level of Phe in the blood. The severity of the disease depends on the serum Phe values. Thus, in relation to the standard values ranging between 10–20 mg/dL (>20 mg/dL or >1.21 × 10^−3^ M [16], the value of 1 mg/ dL (0.061 × 10^−3^ M) indicates benign hyperphenylalaninemia (HPA) and values of 4–10 mg/dL (0.24 0.605 mM) are an indicator of PKU [16].

**Figure 3:**
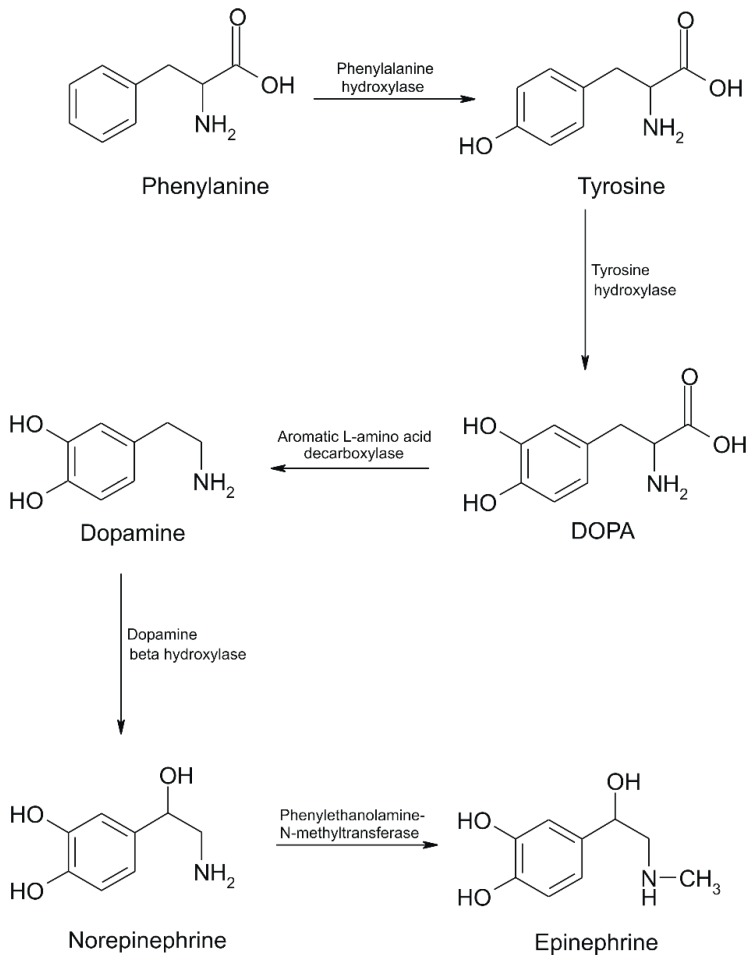
Phe conversion to epinephrine and other catecholamines [16].

In HPA, Phe is transaminated into phenylpyruvate (phenylalanine + α-ketoglutarate = phenylpyruvate + glutamate) instead of tyrosine. The accumulation of phenylpyruvate may lead to mental retardation (respectively PKU) in infants. When Phe is present in excess, it is transaminated with α-ketoglutarate to phenylpyruvate and glutamate, as in PAH deficiency. However, phenylpyruvate formation is insufficient to cause problems in such cases. Moreover, when Phe is in excess, it may be converted into phenylethylamine and other metabolites, such as phenylacetic acid, phenylacetylglutamine and phenyllactic acid [16]. The general conversion of Phe into tyrosine and then catecholamines is shown in Figure 3.

**Figure 4:**
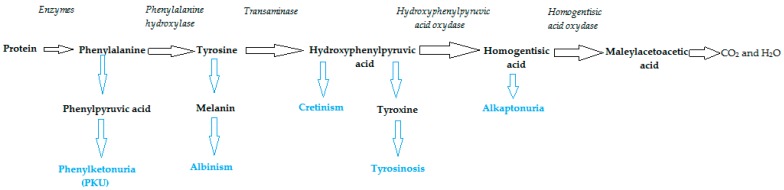
Phe transformation into tyrosine and its metabolism [17].

When Phe conversions to tyrosine and tyrosine metabolites are blocked, various disorders occur in the body. When PAH is not functional, Phe is converted to phenyl pyruvate resulting in PKU. Blocking the conversion of tyrosine into melanin leads to melanin deficiency, at which time the disease called albinism occurs. When the conversion of tyrosine into iodine derivatives is blocked, the thyroid hormone does not develop sufficiently, thus causing cretinism. Tyrosinosis occurs when the conversion of hydroxyphenyl pyruvate into homogentic acid is blocked. Alkaptonuria manifests when the conversion of homogentizic acid into maleylacetoacetate is blocked. Blockages are usually created when the genes of the enzymes involved have undergone such mutations that the protein coding is poorly functional or dysfunctional [17]. These conditions are summarized in Figure 4.

Phe is metabolized in the liver. L-Phe which is not metabolized in the liver, is distributed through the systemic circulation to different tissues of the body, where it undergoes metabolic reactions similar to those occurring in the liver, thus being metabolized in a number of different substances in humans [18,19].

Regarding toxicity, L-Phe exacerbates the symptoms of PKU if used by phenylketonurics. Some studies have shown that L-Phe may aggravate tardive dyskinesia for some schizophrenic patients, but also for those who were given neuroleptic drugs. L-Phe in combination with L-Dopa is dangerous because it impedes the transport of L-Dopa to the brain which may cause major changes in a clinical response known as the “on-off” phenomenon [5].

Phenylethylamine (PEA) is a metabolite of Phe, a neurotransmitter and hormone which can act as a neuromodulator for catecholamines. PEA increases extracellular dopamine levels and modulates noradrenergic transmission. PEA also antagonizes gamma-aminobutyric acid B-receptors (GABABR), suppressing their inhibitory effects. Thus, the supplementation with PEA, together with Phe, are assumed to have antidepressant effects. Different from Phe, PEA use has not been investigated in the pediatric population. A dose of 10–60 mg/day of PEA is generally administered to adults, which corresponds to approximately 0.1–0.7 mg/kg/day [20].

Research studies show that Phe and PEA have been well tolerated by the body, with very few adverse effects, excluding nausea, fatigue, changes in sleep or cardiovascular problems. It was found that both PEA and Phe from supplements should be administered in the morning. No “switching” in manic or hypomanic states were reported [20].

## 3. Analytical Methods for Phe Determination

Various analytical chemical (gravimetric, volumetric) and instrumental (optical, electrochemical, separation, thermal, resonance) methods were used in the present research for determining Phe with the aim of fulfilling performance criteria such as: accuracy, precision, selectivity, sensitivity, limit of detection, duration and cost.

### 3.1. Chemical Methods

Although very few, due to their being expensive and requiring specific analytical skills, the chemical methods applied to determine Phe, which constitute the basis of the following research, include gravimetric methods (inorganic, organic precipitation agents, electrodeposition) and volumetric methods (acid–base titrations, de precipitation, complexonometry, oxido-reduction) [21].

### 3.2. Instrumental Methods

These are the most numerous according to the research carried out so far. In determining the essential amino acid Phe, optical methods were used, respectively: colorimetry [22,23]. UV and IR spectrophotometry [24], fluorescence [25,26,27,28,29], enhanced fluorescence [30], chemiluminescence [31,32,33]; separation methods, such as: gas chromatography [34], capillary electrophoresis, HPLC—high performance liquid chromatography [22,35,36,37,38,39,40,41,42,43,44]; spectroscopic methods: Raman spectroscopy [45,46], laser-assisted spectroscopy [47], UV-Vis spectroscopy and chemometry [48]; UV−Vis and surface-enhanced Raman scattering [49], nuclear magnetic resonance [50] and circular dichroism spectroscopy [51]; mass spectrometry [22,34,41,52], fluorimetry [53].

Although these methods have proven to be efficient, a series of disadvantages could be identified: they are costly, time consuming, and require special analysis and equipment. This explains why the electrical and electrochemical methods, i.e., potentiometry, voltammetry and conductometry, which imply lower costs, accessible maneuverability and allow for higher sensitivity have been preferred in Phe determination recently.

### 3.3. Sensors and Biosensors

Considering the category of instrumental methods, the electrochemical ones, which have developed in recent years, they are based on the construction of sensors or biosensors and measure one of the following features: electrode potential, current intensity through the cell, the amount of electricity passing through the cell, resistance and the time required for the development of the electrode process [21].

Cyclic voltammetry (CV) is one of the methods most frequently used for characterizing electrochemical systems, because it provides both qualitative and quantitative information about a studied system. The graphical representation of the current recorded by the working electrode according to the applied potential is called the cyclic voltammetry curve. Using this method, a variety of sensors and biosensors may be applied, studied and modified so as to determine the substance to be analyzed. Molecular printing is the novelty of these devices, as they have numerous improved features: high sensitivity, short response time, simplicity of electronic interface, portable tools [54,55].

Differential pulse voltammetry (DPV) and square-wave voltammetry (SWV) are pulsed techniques used in sensing and biosensing [56,57]. The excitation potential in SWV consists of a symmetrical square-wave pulse of a fixed amplitude superimposed on a staircase waveform of step height. In this technique the forward pulse of the square wave coincides with the staircase step [58]. DPV is a voltammetric technique, similar to SWV, where the potential excitation consists of small pulses, which are superimposed upon a staircase waveform [59].

The main advantage demonstrated by these techniques is the low capacitive current (background current) which results in the enhancement of the sensitivity of the pulse voltammetric procedures. However, DPV is usually applied in the case of irreversible systems or in systems presenting slow-reaction kinetics. SWV is more often applied for the study of reversible systems (rapid reaction kinetics systems [59,60].

**Figure 5:**
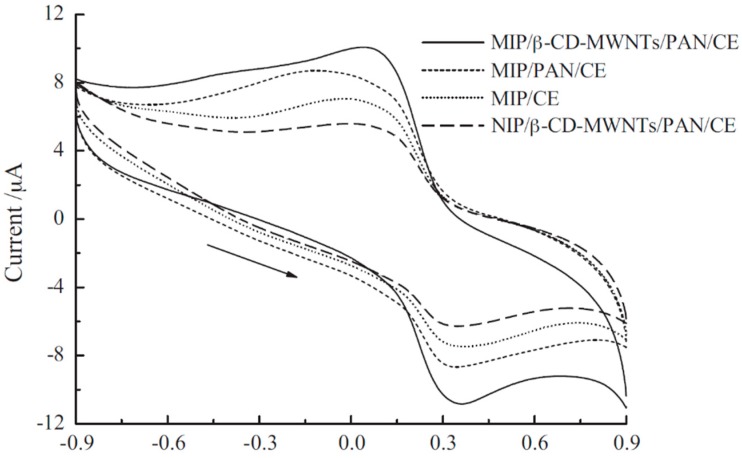
Voltammetric responses of the sensor in different stages of active surface modification immersed in 10^−3^ mol × L^−1^ L-Phe in blood plasma samples. Reprinted from [61] with permission of publisher.

Hu et al. developed a sensitive and selective device, more precisely an electrochemical sensor for the determination of L-Phe from a carbon electrode modified with MIP/*β*-CD-MWNTs/PAN (molecularly imprinted polymer)/*β* – cyclodextrin - multiwalled carbon nanotubes/polyaniline). This sensor has been studied by CV, DPV and amperometry and has been proved to have very good selectivity, stability, sensitivity, reproducibility and analytical recovery for the detection of L-Phe in blood plasma samples [61]. Figure 5 shows the voltammetric responses of the sensor in different stages of the active surface modification (non-imprinted sensor NIP/*β*-CD–MWNTs/PAN/CE) towards L-Phe from blood plasma samples.

As shown in Figure the MIP/*β*-CD–MWNTs/PAN/CE presents the highest peaks in blood plasma samples, which is related to the functional layers involving *β*-CD–MWNTs and PAN. In addition, the peaks of MIP/CE was lower comparing with the MIP/PAN/CE, fact related to the electrical conductivity and catalytic properties of PAN. The voltammetric peaks of NIP/*β*-CD–MWNTs/PAN/CE were lowest because this film lacks in imprinted bind sites being a non-imprinted polymeric film. The improved sensing of MIP/b-CD–MWNTs/PAN/CE towards L-Phe is related to *β*-CD–MWNTs/PAN composite film, which shows excellent stability, high conductibility, good electrocatalytic properties and active bind sites. These factors—combined with specific inclusion interactions between sensitive film and L-Phe assure the enhanced sensitivity and selectivity of this sensor.

**Figure 6:**
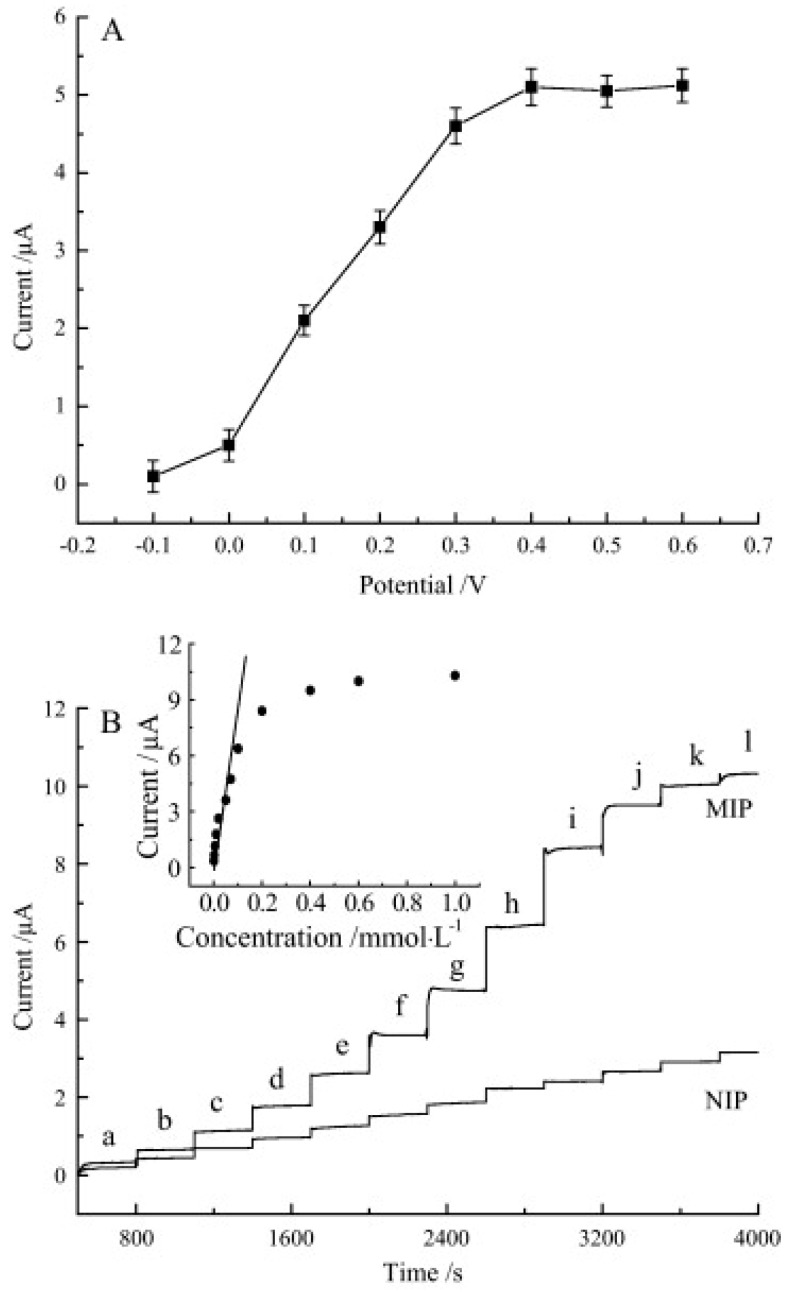
(**A**) Polarization curve of sensor in 10^−4^ mol × L^−1^ L-Phe (electrolyte phosphate buffer solution). (**B**) Chronoamperograms of the MIP/*β*-CD–MWNTs/PAN and NIP/*β*-CD–MWNTs/PAN sensors towards different concentrations of L-Phe; Concentration (mol × L^−1^) of L-Phe are in the range 5.0 × 10^−7^–1.0 × 10^−3^ mol × L^-1^; Inset: Calibration curve of MIP/*β*-CD–MWNTs/PAN sensor towards L-Phe. Reprinted from [61] with permission of publisher.

The polarization curve of the sensor, the amperometric response and the calibration curve are presented in Figure 6.

The detection limit (LOD) obtained is 1.0 × 10^−9^ mol × L^−1^ in the concentration range 5.0 × 10^−7^ at 1.0 × 10^−4^ mol × L^−1^. The sensor response highlighted interaction by inclusion of *β*-cyclodextrin, high PAN conductivity and an excellent molecular recognition ability of the printed film for L-Phe.

Kutyła-Olesiuk et al. quantitatively analyzed 4 amino acids, including Phe, the amino acid under scrutiny in this review. They built sensors from electrodes modified by incorporating phenylboronic acid into the PVC/DOS membrane containing anionic additives (KTFPB), and they obtained the following potentiometric response: a higher sensitivity for Phe and glutamic acid to ornithine and tyrosine, the latter not exceeding 10 mV /decade. All measurements were performed under the following conditions: Ag/AgCl reference electrode (KCl 3 mol × L^−1^), 1 mol × L^−1^ CH_3_COOLi support electrolyte. Data analysis was performed in MatLab and Origin. The chemical images of the samples were processed by using discriminatory analysis solved by partial least squares (PLS-DA). Depending on the experimental pH conditions, anionic (pH = 4) or cationic (pH = 9) responses with slopes <10 mV/decade resulted in a fairly narrow linear range of amino acid concentration (except for Phe responses to pH = 4) [62].

Wang et al. characterized, by using electrochemical impedance spectroscopy (EIS) and CV, an LNT (LaNi_0.5_Ti_0.5_O_3_) - modified carbon electrode (MCE), which represented the starting point in the construction of an electrochemical sensor used for detecting 4 amino acids: L-cysteine (L-Cys), L-tryptophan (L-Trp), L-alanine (L-Ala) and L-Phe. The amino acid sensor showed good reproducibility, long-term stability and fast amperometric response [63]. A low LOD and a high sensitivity were obtained as compared to those previously reported in the domain-specific research. The results demonstrate that LNT/MCE is a good alternative for determining amino acids, therefore the perovskite-type oxide material is a good option for the quantitative determination of amino acids. Table 1 presents the analytical characteristics of the sensor regarding the detection of some amino acids.

The amino acid Phe was further determined in human serum by using an electrochemical biosensor. This constructed biosensor is based on an enzyme-modified Au electrode with functionalized *n*-propylamine (mobile crystalline material 41 (MCM-41-nPrNH_2_), which was subsequently introduced into a phosphate buffer solution with a pH 7.00 for the Phe electrooxidation, indicating that this process is irreversible and diffusion controlled.

**Figure 7:**
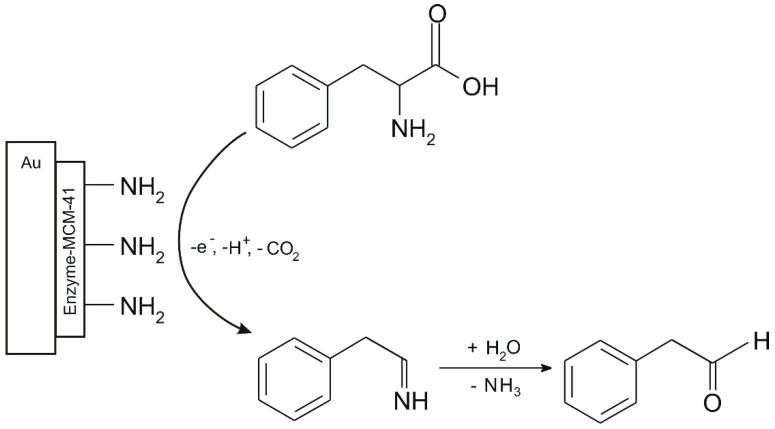
The detection mechanism of the biosensor [64].

The detection mechanism of the biosensor is shown in Figure 7.

The detection mechanism of this enzymatic sensor is related to electrostatic interaction of -NH_2_ functional group from the enzyme MCM-41-nPrNH_2_ with Phe, which facilitate the transport of Phe toward the electrode and the electrochemical reaction. At the same time, by formation of hydrogen bonds with the carboxylic acid functional group of Phe facilitate the oxidation process.

As regards detection methods, CV, DPV, linear sweep voltammetry (LSV) and SWV were used.

The experimental conditions influencing the determination of Phe were optimized and in optimal conditions, the oxidation peak current was proportional to the Phe concentration in the range 0.01–0.15 × 10^−6^ mol × L^−1^, while the LOD was 0.006 × 10^−6^ mol × L^−1^ (S/N = 3).

The results obtained regarding the analytical recovery were satisfactory, with a percentage of 95.1% to 104.2%, the conclusion being that the biosensor has a high sensitivity and a fast response time. The enzymatic sensor exhibits high sensitivity and fast response towards Phe and it can be used as an electrochemical detector of chromatographic and electrophoretic separation systems [64].

In 2014, a simple and sensitive biosensor for Phe detection was developed. The biosensor was made by using a gold electrode and modified with a 5-thiol aptamer and it was called an electrochemical aptasensor. CV and DPV were the methods used to determine Phe, initially in a Tris-HCl buffer solution with physiological pH of 7.4.

**Figure 8:**
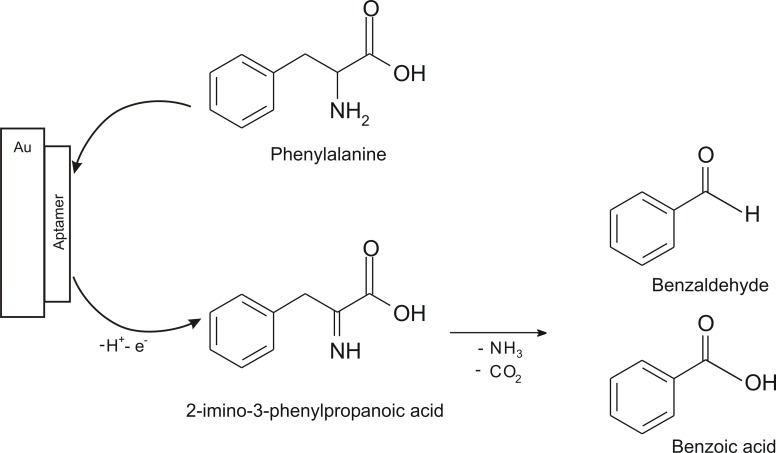
Biosensor detection mechanism [65].

By the immobilization of Phe aptamer onto Au electrode, a selective interaction occurs between sensitive layer and L-Phe. These specific interaction are followed by the electrochemical and chemical processes described in Figure 8.

As presented in Figure 8, from Phe by the transfer of two electrons and two protons an intermediate is formed (2-imino-3-phenylpropanoic acid). By decomposition of the intermediated, when NH_3_ and CO_2_ are eliminated, the benzaldehyde and benzoic acid were formed as main products.

The LOD and the sensitivity of the biosensor to Phe were estimated to 1 × 10^−9^ mol × L^−1^ (S/N = 3), respectively 0.367 μA × nM^−1^. The linearity interval of the signal was observed between 1 and 10 × 10^−9^ mol × L^−1^ Phe with a correlation coefficient of 0.9914. This biosensor was designed, on the one hand, to obtain the most accurate and rapid results in the analysis of proteins from biochemical and biomedical studies and to be used as a voltammetric detector of Phe in flow systems when coupled to chromatography and electrophoretic separation systems, on the other [65].

In 2015, [66] developed a biomimetic sensor built from a molecular imprinted electrode with a hydrophobic-hydrophilic 2:1 combination of EDMAA (ethyleneglycol dimethacrylate) and MBAA (methylene bisacrylamide) which proved to be sensitive to an aqueous Phe solution. CV was used as the detection method, where the dynamic range of the current was from 0.2 to 1 × 10^−3^ mol × L^−1^ (with R^2^ > 0.95) and changes of the current were observed in the concentration range of the Phe solution of 3–5 × 10^−6^ mol × L^−1^.

**Figure 9:**
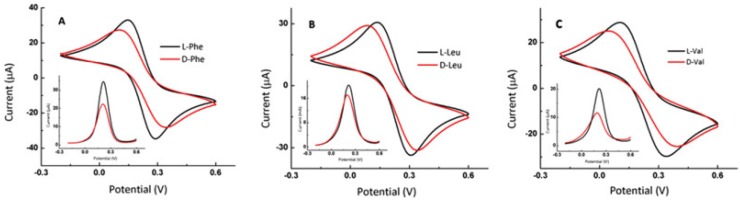
CV and DPV signals of D-Phe (5 × 10^−3^ mol × L^−1^) and L-Phe (5 × 10^−3^ mol × L^−1^) (**A**), D-Leu (5 × 10^−3^ mol × L^−1^) and L-Leu (5 × 10^−3^ mol × L^−1^) (**B**) and D-Val (5 × 10^−3^ mol × L^−1^) and L-Val (5 × 10^−3^ mol × L^−1^) (**C**) at TOCNC/L-Cys/Au electrodes in 5 × 10^−3^ mol × L^−1^ [Fe (CN) _6_]^4–/3–^ solution with 0.1 mol × L^−1^ KCl. Reprinted from [67] with permission of publisher.

Bi et al. manufactured an electrochemical sensor for the detection and identification of the amino acid enantiomers: Phe, leucine and valine, markers of metabolic diseases. This sensor was based on electrodes made of TOCNC (oxidized cellulose nanocrystals)/L-Cys/Au [67]. CV and DPV were the methods used to study the TOCNC/L-Cys/Au electrode. The voltammograms obtained are shown in Figure 9.

As can be observed in Figure 9, when the L- and D-Phe were present in solution, the oxidation and reduction peak currents observed in cyclic voltammograms decreased compared to blank solution. It can be related to lack of electroactivity of Phe, which difficult the transfer of electrons. The same situation happens for the curves registered by DPV. Furthermore, the peak current of D-Phe was lower than L-Phe, the ratio between the currents of the two enantiomers being 1.55 for the TOCNC/L-Cys/Au electrode in DPV. This prove that the TOCNC/L-Cys/Au electrode selectively detect Phe enantiomers.

Additionally, the TOCNC/L-Cys/Au electrode could selectively recognize Leu and Val enantiomers, both by CV and DPV. However, no difference between peak potentials was observed only for Phe enantiomers. Therefore, this sensor and the DPV method fit better for the detection of the L- and D-Phe.

The TOCNCs were previously characterized by using the Fourier transform infrared spectroscopy (FTIR) and the scanning electron microscopy (SEM). The modified electrodes were characterized by SEM and by electrochemical techniques. Considering the DPV results, the peak currents of the two enantiomers were shown to decrease linearly according to their concentrations. This type of electrode showed a stronger adsorption of amino acids D than of amino acids L, creating a clear difference between the peak currents corresponding to amino acids L and D. On actual samples, differences were observed between the voltammetric responses of the serum samples in the case of healthy people as compared to those of type 2 diabetes patients, a significant difference being noticed in the modified electrode. This sensor is useful for screening, diagnosing and treating multiple metabolic diseases.

**Figure 10:**
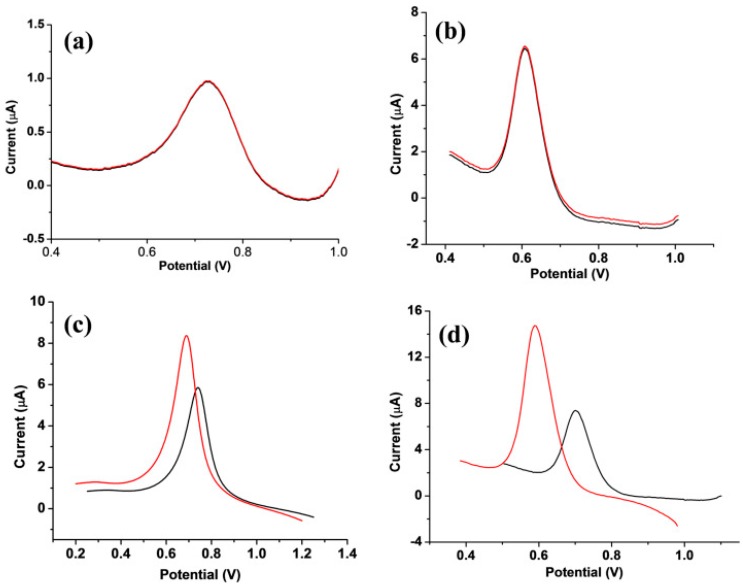
DPV responses of (**a**) bare GCE, (**b**) RGO/GCE, (**c**) *β*-CD/GCE and (**d**) *β*-CD/RGO/GCE in the presence of L-Phe, (red) and D-Phe (black) in 0.1 M PBS (pH 7.0) at scan rate of 100 mV × s^−1^. Reprinted from [68] with permission of publisher.

Zaidi developed a useful sensor for the enantiomeric recognition of Phe. A glassy carbon electrode modified with *β*-CD (*β*-cyclodextrin) immobilized on reduced graphene sheets (RGO-reduced graphene oxide) was prepared [68]. The proposed sensor was studied by using the following methods: SEM, FTIR and EIS techniques. Analytical studies showed that *β*-CD/RGO/GCE has superior chiral recognition in relation to L-Phe than D-Phe. The results obtained by DPV are shown in Figure 10.

Figure 10 shows the response of the electrode in different stage of the chemical modification. In the case of GCE no difference between the peaks related to Phe enantiomers was observed (Figure 10a). The DPV curves of RGO/GCE showed an increasing of the peaks, but the differences between Phe enantiomers is not achieved (Figure 10b). The signals registered with *β*-CD/GCE showed differences bot as current and potential, the fact being related to formation of inclusion complexes between both Phe enantiomers and *β*-CD (Figure 10c). *β*-CD exhibit different affinity for the enantiomers, and as can be observed in Figure the detection of L-Phe is better, the peak is higher, and the potential is lower. The differences between the voltammetric curves of *β*-CD-RGO/GCE (Figure 10d) towards enantiomers of Phe are highest. There are two synergic effects that increase the detection capacity: formation of inclusion complex with *β*-CD more favorable for the L enantiomer and the favorization of the electron transfer by RGO.

Under optimal conditions, the developed sensor has a good linearity range between 0.4 and 40 × 10^−6^ mol × L^−1^ and LODs with values of 0.10 × 10^−6^ mol × L^−1^ for L-Phe and 0.15 × 10^−6^ mol × L^−1^ for D-Phe. The proposed sensor has good stability and regeneration capacity.

**Figure 11:**
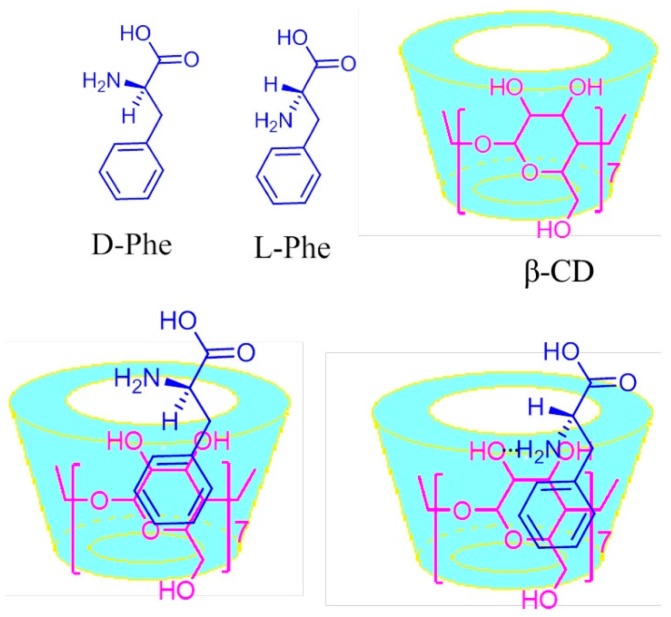
Formation of the inclusion complexes of chiral isomers of Phe with *β*-CD. Reprinted from [73] with permission of publisher.

The recognition mechanism of Phe enantiomers by means of the formation of the inclusion complexes with *β*-CD, present in the case of sensors above described, are illustrated in Figure 11.

As can be seen in Figure 11, when the phenyl functional group of the Phe enters into the *β*-CD inner cavity, the inclusion complex can be formed. However, the amino functional group (-NH_2_) is on the *β*-CD opening edge. The hydrogen bond between the L-Phe (-NH_2_) and the *β*-CD (-OH) is stronger than that with D-Phe, and therefore the electrochemical detection of L-Phe is more sensitive (higher peaks, lower potential) [68].

A new type of electrochemical sensor, which has proven reliable, was developed to determine the amino acid L-Phe by molecular imprinting, respectively by electropolymerization on the Au electrode in the presence of L-Phe. The template molecule used and printed with TP3C-Trp (thiophene-3-carbonyl tryptophan) was L-Phe. In addition to the CV, the studies for the characterization of the sensor were carried out by using methods, such as: atomic force microscopy, FTIR, measurement of ellipsometry and contact angle. The linearity range and the LOD of the developed sensor were 1.0 × 10^−8^–1.0 × 10^−7^ mol × L^−1^ and 1.0 × 10^−9^ mol × L^−1^, respectively, thus showing a low LOD and high selectivity for the determination of L-Phe. This built-in sensor exhibited good recognition ability for the template molecule as compared to other molecules with similar structures [5].

In 2017, a DNA-based electrochemical biosensor was designed for the rapid and sensitive determination of a genetic mutation (IVS10nt546) linked to the enzyme PAH, the leading cause of PKU, a metabolic disease found more often nowadays. The biosensor is built from a gold electrode printed and modified with thiol groups, which uses hematoxylin as an electrochemical indicator. CV and EIS were the methods used and they proved to be efficient in solution of [Fe(CN)_6_] ^3−/4−^. Under optimal conditions, the biosensor was able to detect the target DNA resulting in a wide dynamic range between 2 × 10^−11^ mol × L^−1^–1.5 × 10^−7^ mol × L^−1^, a significantly lower LOD of 8.5 × 10^−6^ mol × L^−1^ and good results in the analysis of the real samples. The biosensor can be used simply and quickly in biomedical analysis for the early detection of PKU [69].

In the same year, 2017, a molecular electrochemical sensor was developed for the enantiomeric recognition of L-Phe, this sensor being successfully used in drug analysis and bioanalysis. Molecular printing was performed with a polymer, an organometallic complex (MOF) deposited on a gold electrode in the presence of L-Phe and a functional monomer (4-aminophenol) by electropolymerization, using thiolated *β*-CD and L-cysteine. The methods selected to characterize the modified electrode were CV and EIS, using the hexacyanoferrate solution as a sample with redox properties. The LOD obtained with this sensor was 3.3 × 10^−13^ mol × L^−1^, a limit which is a much lower than what had been reported in the case of other electrodes. Different from D-Phe, the modified electrode showed a much better selectivity for L-Phe at 0.2 V vs. Ag/AgCl, having a recognition coefficient of 2.12 than D-Phe, due to the organometallic complex and the specific interaction of L- Phe with *β*-CD. The sensor was successfully applied to L-Phe analysis in urine [70].

**Figure 12:**
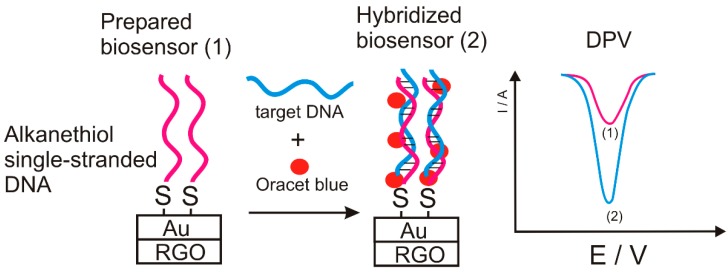
Nano-biosensor detection scheme.

In order to detect PKU, Seifati et al. developed, in 2017, an electrochemical nano-biosensor constructed from gold nanoparticles decorated on reduced graphene oxide (RGO) immobilized on screen-printed carbon electrode. A specific alkanethiol single-stranded DNA probe was attached, which interacts specifically with specific target DNA, and the Oracet blue as an electrochemical label [71]. The biosensor detection scheme is shown in Figure 12.

The biosensor detection mechanism is based on the hybridization of the target DNA sequence with the complementary DNA probe. The selectivity and specificity of the biosensor was very good making a clear difference between single strand DNA and target hybridized double-strand DNA. Furthermore, the difference between target DNA with non-complementary DNAs is successful achieved.

CV and microscopy were used to determine the Phe level and to determine a PAH mutation. The limit of detection obtained was 21.3 × 10^−15^ mol × L^−1^, and the dynamic range was 80–1200 × 10^−15^ mol × L^−1^. Good specificity and sensitivity were obtained for the target DNA than other specific sequences such as double hybridized DNA. The nano-biosensor proved to be an easy-to-use, low-cost and optimally optimized biosensing platform in various fields of research, for example, in medical, biochemical and food industries.

Lin et al. developed a biosensor based on biologic cells for the detection of Phe and tyrosine, this biosensor being used for testing real samples, more precisely urine samples, and proving a precise instrument for detecting metabolic diseases, such as PKU. The experimental methods used were liquid chromatography-mass spectrometry and fluorescence spectroscopy. The LOD for Phe was 3.7 × 10^−6^ mol × L^−1^ and the linearity range was 5 × 10^−6^–100 × 10^−6^ mol × L^−1^, with R^2^ = 0.996 [72].

The single-use electrochemical microsensor modified with a PtPd@ZIF-67 nanocomposite (imidazole-modified zeolite) is a novelty in the research studies regarding the determination of Phe level. When using electrochemical methods, the microsensor exhibited selectivity to phenylpyruvic acid and phenylacetic acid, different from the response to other analogous amino acids where no positive results were obtained. The FTIR method was also used in this study to detect the acylation reaction between imidazole from ZIF-67 and carboxyl from PKU markers, ensuring chemoselectivity. The electrochemical performances were due to the encapsulation of the PtPd alloy in nanoparticles of ZIF-67, which facilitates the electron transfer. Another advantage of this microsensor is that it can distinguish PKU markers in the presence of other analogous amino acids and other physiological compounds or ions, demonstrating that it has a good selectivity and that interferences are reduced.

The results obtained with this modified sensor, PtPd @ ZIF-67, such as good specificity and rapid response to PKU markers, are promising for the early detection of PKU [3].

Using different electrochemical methods: i.e., CV, SWV and DPV [13] developed a sensor based on ProDOT-Boc-Phe ((3,4-dihydro-2H-thieno [3,4-b] [1,4] dioxepin-3-yl) methyl 2—[(tert-butoxycarbonyl) amino) glassy carbon electrode ] -3-phenylpropanoate)—which was prepared by electrochemical polymerization for the rapid and efficient chiral recognition of DOPA enantiomers (L-DOPA and D-DOPA), showing good stability and reversible redox activities. The SWV resulted in a recognition efficiency of 1.28 for (D) -PRODOT-Boc-Phe and 1.17 for (L) -ProDOT-Boc-Phe.

Considering that the electrochemical graphene sensors modified with cyclodextrin have been widely used in chiral recognition, Niu et al. developed, via CV and DPV, a chiral electrochemical sensor constructed from an electrode modified with rGO-Fc-CD (oxide de graphene-ferrocene- *β*-cyclodextrin) for the enantiomeric recognition of L- Phe and D- Phe [73]. FTIR, XRD, TGA, SEM and XPS were the methods used in the synthesis and characterization of the compounds. The chiral recognition capacity was higher in the case of L-Phe than D-Phe and the enantioselectivity coefficient (ID/IL) of the electrochemical sensor, under optimal conditions, was 2.47. The LOD reached values of 2.7 × 10^−8^ mol × L^−1^ for L-Phe and 5.2 × 10^−8^ mol × L^−1^ (S/N = 3) for D-Phe, the linearity range being 0.01–5.0 × 10^−6^ mol × L^−1^.

The feasibility of the sensor was evaluated on real samples, respectively on serum samples taken from adults and infants, with and without PKU and the results varied: for adults from 3.0% to 4.7% and the analytical recovery varied from 98.8% to 100.9%; in infants the values ranged from 3.5% to 5.0% and the analytical recovery ranged from 98.7% to 101.8%, confirming the good sensitivity of the modified chemical electrochemical sensor rGO-Fc-CD/GCE.

The same authors, [12], developed another electrochemical sensor based on a modified electrode rGO-PTCA-CD (graphene oxide—3,4,9,10-perylene tetracarboxylic acid—cyclodextrin). The methods used for the synthesis and characterization of the compounds were FTIR, SEM, X-ray spectroscopy and the electrochemical method. By means of CV and DPV, the working electrodes which used [Fe(CN)_6_]^4−/3−^ as the redox reference sample and ferrocene as the standard interference sample, were analyzed and used for Phe recognition. The enantioselectivity coefficient of the modified glassy carbon electrode rGO-PTCA-CD was 2.07 in the case of L-Phe. The LODs obtained for L-Phe and D-Phe were 8 × 10^−5^ mol × L^−1^, respectively 2 × 10^−10^ mol × L^−1^ (S/N = 3) and the linear response ranged between 1 × 10^−5^–0.05 mol × L^−1^. The results showed that the use of this biosensor has the following advantages: excellent reproducibility, stability, enantioselectivity for the recognition of Phe enantiomers, low cost, high efficiency. On real samples (urine and blood serum), the proposed biosensor proved to be feasible, the analyzes carried out leading to the following results: the relative standard deviation (RSD) between 3.1% and 5.2%, the analytical recovery from 98.5% to 102.5% for L-Phe detection; for D-F RSD detection ranged from 3.6% to 6.1% and the analytical recovery ranged from 98.5% to 101.3%.

Sajini et al. built a new sensor based on photo-reactive molecular printed polymer, for the specific and selective recognition of the L-PABE (L-Phe benzyl ester) molecule. The working electrode of the sensor is made of platinum modified with multilayer carbon nanotubes and the molecular printed polymer prepared from L-PABE and MPABA (benzoic acid 4—[(4 methacryl biloxi) phenylazo]), using as a cross-linking agent EBMAA (***N, N***-ethylene bismetacrylamide). CV, DPV and EIS were the detection methods used for the sensor study. The LOD of the sensor was 0.2086 × 10^−6^ mol × L^−1^ and the limit of quantification was 0.6953 × 10^−6^ mol × L^−1^. The highest absorption capacity for L-PABE, compared to its analogous enantiomers, was presented by MWCNT-MIP (multiwalled carbon nanotubes) and MIP (molecularly imprinted polymers). The sensor has proved to be efficient because L-PABE adsorption on MWCNT-MIP is an adsorption which adjusts to the Langmuir isotherm and thermodynamic studies have shown that adsorption is controlled by enthalpy. These MWCNT-MIPs had enthalpies and entropies higher than conventionally printed polymers and contributed to specific recognition [74].

**Figure 13:**
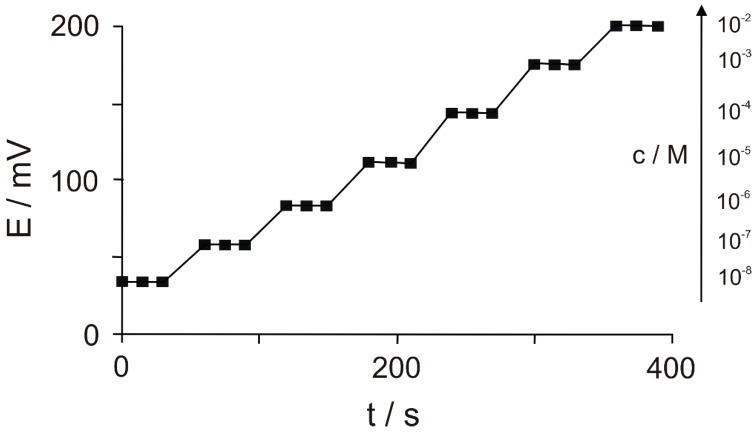
Potentiometric sensor response immersed in Phe solutions of different concentrations [7].

Bangaleh et al. developed a potentiometric sensor to determine blood serum Phe from a molecularly imprinted graphite electrode with a mixture of polyvinyl chloride and a plasticizer dissolved in THF (tetrahydrofuran). The LOD obtained was 5 × 10^−9^ mol × L^−1^, the linearity range of the sensor response being from 1 × 10^−8^ to 1 × 10^−4^ mol × L^−1^ and with a Nernstian response under optimal conditions of 29.73 ± 1.0 mV decade^−1^ [7]. The potentiometric sensor response is shown in Figure 13.

As can be observed, the potentiometric sensor reaches to its steady-state response in less than 20 s in all solutions of Phe with different concentrations.

The actual samples used for this sensor were blood serum, and the results were compatible with those obtained by using the HPLC method. The sensor proved fast, cost effective, easy to use and sensitive for the detection of Phe in biologic liquids.

Finally, the sensitive materials and the analytical features for the principal electrochemical sensors and biosensors used for Phe detection, most of them detailed above in this review, are summarized in Table 2.

## 4. Conclusions

The present review made an inventory of the most recent researches for Phe detection by using electrochemical sensors and biosensors. Electrochemical sensors and biosensors have attractive analytical characteristics and may become useful tools in clinical diagnosis due to the fact that their determinations are simple, fast, sensitive and selective.

Sensitive materials used to construct (bio)sensors are in particular (bio)nanocomposites, hybrid nanomaterials and 3D nanomaterials. The main interest was focused on the use of molecular imprinted polymers, followed by hybrid sensitive materials and less in aptamers or enzymes. By using such sensitive materials in the modification of classical electrodes, high quality analytical performances were obtained. However, the major challenge in this area remains the analysis of the real samples, because reproducibility, stability and non-interferences in the case of determinations with (bio)sensors are very necessary. These objectives can be obtained by developing novel sensors based on chiral nanostructured electroactive materials, which can allow a selective detection of the Phe enantiomers. Superior analytical performances could be obtained by the combination of the enzymes with nanomaterials and electroactive electron mediators. The detection of L-Phe is more studied comparing with D-Phe, even both isomers are important for the human body. Therefore, it is expected the development of novel (bio)sensors able to selectively detect D-Phe. The inclusion of Phe isomerase in the sensitive layer could be useful for facilitate the detection of one enantiomer from a mixture.

Most (bio)sensors based on electrochemical detection methods present a rapid, selective and highly sensitive analysis of Phe in biologic systems, without involving stages of sample preparation to be analyzed. Thus, (bio)sensors are useful analytical tools which may be applied in clinical analysis.

A critical view of the state of the art in the detection of phenylalanine field, evidently opens novel possibilities for electroanalytical tools towards fast screening-based diagnostics, opening new ideas in the PKU diagnosis and monitoring. Therefore, the next stage into the future would be to carry out more analysis using positive clinical samples and validate the electroanalytical methods with standard method for Phe. Another future development direction in electrochemical biosensitivity will be the development of implantable sensors for continuous health monitoring. For this purpose, new (bio)nanomaterials need to be integrated into specific devices so as to achieve good long-term stability and limit contamination. New detection techniques, such as ultra-fast CV or electrochemical scanning microscopy should be implemented for real-time Phe monitoring.

It is reasonable to expect in the near future the development of commercial systems for Phe screening analysis based on (bio)sensors as well as detector systems compatible with chromatographic or electrophoretic systems.

## Figures and Tables

**Table 1:** The analytical characteristics of the sensor regarding the detection of amino acids: L-Cys, L-Trp, L-Ala and L-Phe. Reprinted from [63] with permission of publisher.

Amino Acids	Structure	Oxidated Amino Acids	Linear Range (μM)	R	Sensitivity (nA/μM)	Detection Limit (μM)
L-Cys	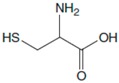	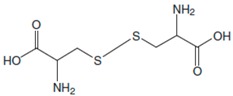	2.5–40	0.998	55	0.5
L-Trp	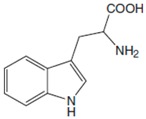	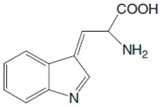	5–60	0.996	38.2	0.67
L-Ala	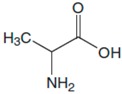	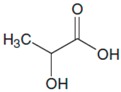	50–600	0.997	3.08	8
L-Phe	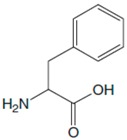	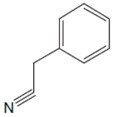	25–500	0.997	3.84	3

**Table 2:** Main electrochemical sensors and biosensors used for Phe detection.

Sensitive Material	Electroanalytical Technique	LOL (mol × L^−1^)	LOD (mol × L^−1^)	Ref.
ZIF-67 Encapsulated PtPd Alloy Nanoparticle (PtPd@ZIF-67)	CV, CA	5–500 × 10^−6^	20 × 10^−9^	[3]
MIP–Thiophen-3-carbonyl tryptophan	CV	1.0 × 10^−8^–1.0 × 10^−7^	1.0 × 10^−9^	[5]
MIP polyvinyl chloride	Potentiometry	1 × 10^−8^–1 × 10^−4^	5 × 10^−9^	[7]
Perylene-functionalized graphene/β-CD	CV, DPV	0.01–5 × 10^−3^	0.08 × 10^−9^ for L-Phe 0.2 × 10^−9^ for D-Phe	[12]
MIP/*β*-CD-MWNTs/PAN	CV, DPV, CA	5.0 × 10^−7^–1.0 × 10^−4^	1.0 × 10^−9^	[61]
Phenylboronic acid/PVC	potentiometry	0.1–3 × 10^−3^	-	[62]
Perovskite nanoparticles (LaNi_0.5_Ti_0.5_O_3_)	CV, EIS	25–500 × 10^−6^	3 × 10^−6^	[63]
Phenylalanine Dehydrogenase /Amino-Functionalized Mobile Crystalline Material-41	CV, DPV, SWV, LSV	0.01–0.15 × 10^−6^	0.006 × 10^−6^	[64]
5′ Thiolated L-Phe aptamer/Au	CV, DPV	1–10 × 10^−9^	1 × 10^−9^	[65]
MIP/ethyleneglycol dimethacrylate–methylene bisacrylamide	CV	0.02–1 × 10^−3^	3–5 × 10^−6^	[66]
2,2,6,6-tetramethylpiperidine-1-oxyl-oxidized cellulose nanocrystals/L-cystines/ Au	CV, DPV	0.05–5 × 10^−3^	5.6 × 10^−6^ for L-Phe 9.0 × 10^−6^ for D-Phe	[67]
*β*-CD/RGO/GCE	DPV	0.4–40 × 10^−6^	0.10 × 10^−6^ for L-Phe 0.15 × 10^−6^ for D-Phe	[68]
DNA (thiol modified oligonucleotide probe)/hematoxylin	CV	20 × 10^−12^–1.5 × 10^−7^	8.5 × 10^−12^	[69]
MIP/thiolated *β*-CD/L-cysteine	CV, DPV	2×10^−12^–6×10^−10^	0.33 × 10^−12^	[70]
gold nanoparticles/rGO/alkanethiol single-stranded DNA/Oracet blue	CV	80 × 10^−15^–1200 × 10^−15^	21.3 × 10^−15^	[71]
*E. coli* DH5a cells	FS	5 × 10^−6^–100 × 10^−6^	3.7 × 10^−6^	[72]
Graphene-ferrocene functionalized CD	DPV	0.01–5.0 × 10^−3^	27 × 10^−9^ for L-Phe 52 × 10^−9^ for D-Phe	[73]
MIP (4-[ (4-methacryloyloxy) phenylazo] benzoic acid)/MWCNT	CV, DPV	0.5–3 × 10^−6^	0.2086 × 10^−6^	[74]
Crosslinked polymethylacrylic acid–polycarbazole hybrid MIP	Potentiometry	2.5 × 10^−6^–2.5 × 10^−2^	1.37 × 10^−6^	[75]
β-CD/CNTs@rGO	CV, DPV	0.2–13.0 × 10^−6^	0.08 × 10^−6^	[76]
DNA aptamer/methylene blue	SWV	9.0 × 10^−8^–7.0 × 10^−6^	0.4 × 10^−6^	[77]
Bamboo charcoal–carbon nanosphere	CV, DPV, SWV, LSV	1–100 × 10^−6^	1× 10^−6^	[78]
L-Phe-MIP PPy/Ag	CA	0.1–50 × 10^−3^	1.39 × 10^−3^ for L-Phe 27.7 × 10^−3^ for D-Phe	[79]
Thiolated-CD/ferrocene-coated gold nanoparticles	CV	0.1–1 × 10^−6^	8.4 × 10^−8^	[80]

^1^ CA = chronoamperometry; FS = fluorescence spectroscopy; PPy = polypyrrole.
